# Effects of Intra-Aortic Balloon Counterpulsation Pump on Mortality of Acute Myocardial Infarction

**DOI:** 10.1371/journal.pone.0108356

**Published:** 2014-09-30

**Authors:** Liwen Ye, Minming Zheng, Qingwei Chen, Guiqion Li, Wei Deng, Dazhi Ke

**Affiliations:** 1 Department of Geriatrics Cardiology, the Second Affiliated Hospital of Chongqing Medical University, Chongqing, China; 2 Chongqing Ophthalmology Research Center for the Senile, the Second Affiliated Hospital of Chongqing Medical University, Chongqing, China; 3 Department of Ophthalmology, the Second Affiliated Hospital of Chongqing Medical University, Chongqing, China; University of Tampere, Finland

## Abstract

**Background:**

Several randomized controlled trials (RCTs) have evaluated the effect of intra-aortic balloon counterpulsation pump(IABP) on the mortality of acute myocardial infarction (AMI).

**Objectives:**

To analyze the relevant RCT data on the effect of IABP on mortality and the occurrence of bleeding in AMI.

**Data Sources:**

Published RCTs on the treatment of AMI by IABP were retrieved in searches of Medline, EMBASE, Cochrane and other related databases. The last search was conducted on July 20, 2014.

**Study Eligibility Criteria:**

Randomized clinical trials comparing IABP to controls as treatment for AMI.

**Participants:**

Patients with AMI.

**Synthesis Methods:**

The primary endpoint was mortality, and the secondary endpoint was bleeding events. To account for to heterogeneity, a random-effects model was used to analyze the study data.

**Results:**

Ten trials with a total population of 973 patients that were included in the analysis showed no significant difference in 2-month mortality between the IABP and the control groups. The 6-month mortality in the IABP group was not significantly lower than in the control group in the four RCTs that enrolled 59 AMI patients with CS. But in the four that enrolled AMI 66 patients without CS, the data showed opposite conclusion.

**Conclusions:**

IABP cannot reduce within 2 months and 6–12 months mortality of AMI patients with CS as well as within 2 months mortality of AMI patients without CS, but can reduce 6–12 months mortality of AMI patients without CS. In addition, IABP can increase the risk of bleeding.

## Introduction

Intra-aortic balloon counterpulsation pump(IABP) can increase blood flow in the coronary artery and the brain while reducing afterload and cardiac oxygen consumption [1]. Kantrowitz [2] first reported the clinical application of IABP. Because IABP can quickly improve the effect of patients' clinical symptom, so this technology causes people's attention. Although numerous percutaneous circulation support equipments are applied, the effect of IABP in adjuvant therapy of AMI patients is still controversial. Some previous studies show that IABP benefits for high-risk patients with AMI, but most of these studies are non-randomized and retrospective, with serious selection bias and poor credibility. However, in some other studies, the effectiveness of IABP in adjuvant therapy for severe patients with AMI has not been ensured [14], [18].

The American College of Cardiology and American Heart Association (ACC/AHA) guidelines recommend IABP for patients with unstable angina and non-ST-elevation myocardial infarction UA/NSTEMI with severe ischemia [3]. However, the curative effect of IABP as a treatment of AMI is still unresolved, and it is not clear whether time and cardiogenic shock (CS) influence its effectiveness. To evaluate the curative effect systematically, we performed a meta-analysis of randomized controlled trials (RCTs) on the treatment of AMI by IABP.

## Methods

### 1. Data sources and search strategy

theThe Medline, EMBASE, Cochrane databases, and related websites were searched without restriction by publication date or publication status; however, only articles published in English were selected. The search keywords included intra-aortic balloon counterpulsation, intra-aortic balloon pump, percutaneous coronary intervention, acute coronary syndrome, acute myocardial infarction, IABP, IABC, PCI, and ACS. The last search was conducted on July 20, 2014.

### 2. Selection criteria

To ensure the quality of the meta-analysis, the following selection and exclusion criteria were applied to assess the RCTs that were retrieved during the searches. Only published RCTs that enrolled AMI patients treated with drugs or PCI (percutaneous coronary intervention) were eligible. Only studies of IABP as an intervention for circulatory support were included, and only if the methods, study dates, sample size, and results were clearly and completely described. Trials that were non- or incompletely randomized, that included patients with coronary syndromes other than AMI, or patients treated with coronary artery bypass grafting, that compared IABP with other percutaneous circulation support equipment, or with unclear data reporting were excluded.

### 3. Data extraction and management

In the selected articles, all-cause mortality was the primary endpoint used to assess the curative effect of IABP on AMI. Bleeding was a secondary endpoint used to assess IABP safety. The patients in the selected RCTs were divided into subgroups whether with or without CS to account for treatment effects depending on the patients' condition. Mortality was evaluated in two subgroups depending on follow-up duration: mortality within 2 months (i.e., in-hospital, at 1 month, and at 2 months), and mortality within 6–12 months.

### 4. Methodology/quality assessment

The RCT quality was assessed independently by two reviewers. The 12 RCTs that were selected were assessed using the Cochrane Collaboration bias risk tool, which considers the following six criteria: proper random sequence generation, concealment of subjects' group allocation, blinding during outcomes assessment, complete recording and reporting of outcomes data, and lack of experimental bias.

### 5. Statistical analysis

The data were analyzed using Review Manager 5.1. Endpoints were treated as dichotomous outcomes, and odds ratios (ORs) with 95% confidence intervals (CIs) were taken as a statistical indicator of the curative effect and safety of IABP in AMI. When the event of interest did not occur, the treatment effect of that study was treated as not estimable [9].

The Breslow–Day χ^2^ test (*p*<0.1) and the *I* ^2^ statistic were used to test heterogeneity of the 13 included studies. An *I* ^2^ of less than 25%, indicated low, 25%<*I* ^2^<50% moderate, *p*>0.1; and *I* ^2^>50, a high degree of heterogeneity [4]. When *I* ^2^<50%, a fixed-effects Mantel-Haenzel model was used to analyze the data, and when *I* ^2^>50%, the DerSimonian and Laird random-effects model was found better than the former model. Publication bias was evaluated using funnel plots [5].

## Results

### 1. Study sample selection characteristics

**Figure 1:**
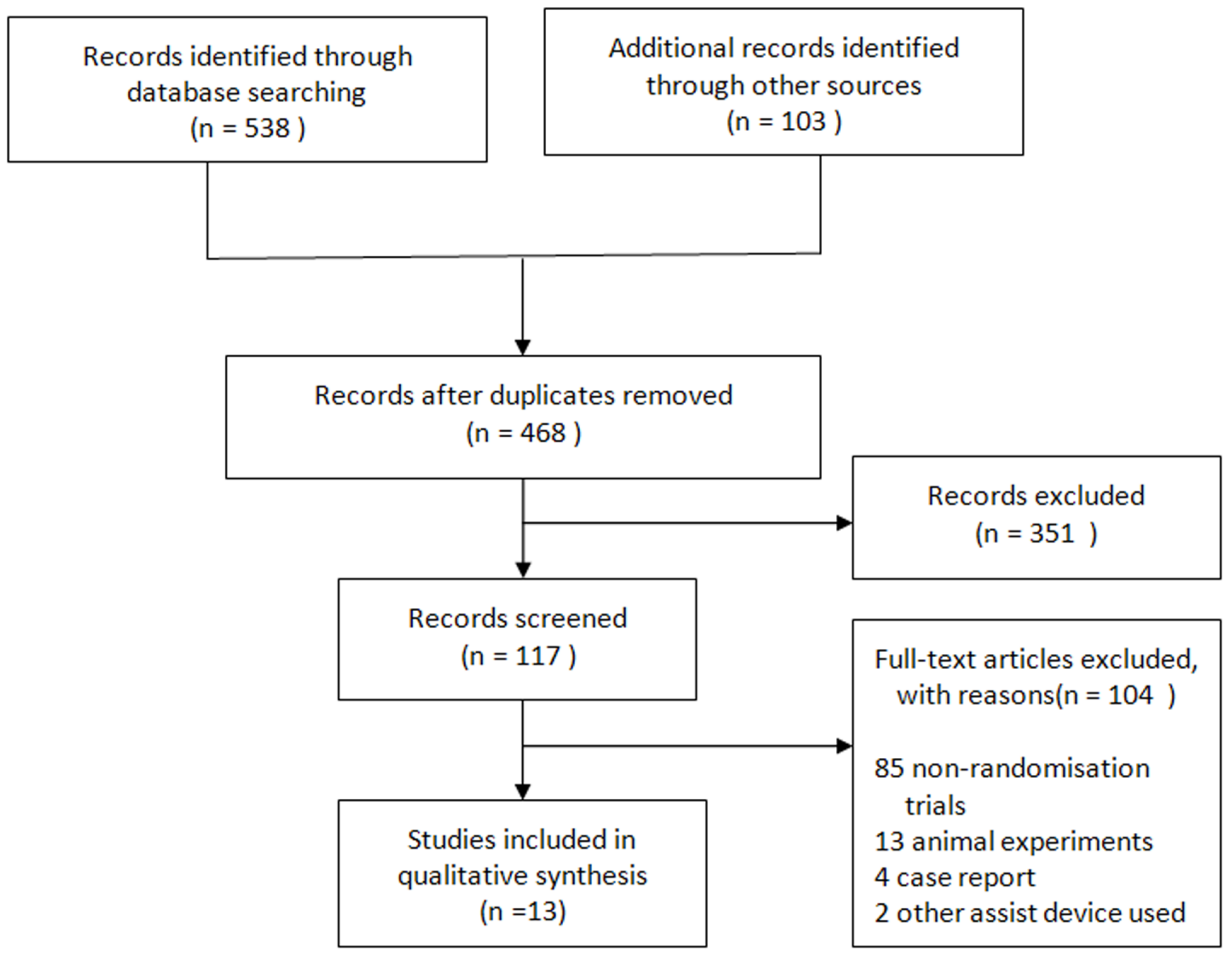
Meta-Analyses statement for trial selection.

A total of 641 potentially eligible publications were retrieved during the searches, and 524 articles not associated with treatment of AMI with IABP were excluded by browsing the title or abstract (Figure 1). After further screening, an additional 104 articles were excluded, including 86 non-RCTs, 13 animal experiments, four case reports, and two articles involving other assist devices. The remaining 13 articles satisfied the selection criteria. The 13 studies enrolled 2237 AMI patients; 1112 were treated with IABP (the treatment group) and 1125 patients were not treated with IABP (the control group). The characteristics of the 13 articles are shown in Table 1. In four of the studies, participants in the IABP were AMI patients with CS; however, in the other eight studies, the patients did not have CS.

**Table 1:** Characteristics of the included articles.

Author(Year)	Patients(n)	Gender(male(%))		Mean age(y)		Inclusion criteria	Adjunctive therapy		IABP after AMI(h)	IABP duration(h)	Follow-up time duration(d)
		IABP	Control	IABP	Control		IABP	Control			
Flaherty 1985 [7]	20	9(90.0)	9(90.0)	52.0	52.0	AMI without CS	IABP+TT	TT	5.0±2.6	34±25	60
Gu 2011 [8]	106	29(56.9)	36(65.5)	67.4	66.6	AMI without CS	IABP+PCI	PCI	5.3±1.9	52±17	30;180
Kono 1996 [9]	45	20(87.0)	16(72.7)	54.0	60.0	AMI without CS	IABP+PTCA	PTCA	2.5±0.5	48	21
Li 2007 [10]	39	12(60.0)	12(63.1)	67.4	64.9	AMI with CS	IABP+PCI	PCI	NR	72	360
Ohman 1994 [11]	182	73(75.6)	64(74)	56.0	55.0	AMI without CS	IABP+TT	TT	<24	12	9
Ohman 2005 [12]	57	23(77.0)	20(74.0)	68.0	67.0	AMI with CS	IABP+PCI	PCI	0.5(0.3–0.9), AR	34(24–68)	30;180
Patel 2011 [13]	337	132(82.0)	144(81.8)	56.1	57.7	AMI without CS	IABP+PCI	PCI	3.4(2.4–4.5)	22.1 (16.8–26.1)	180
Perera 2010 [14]	301	122(81.0)	117(78.0)	71.0	71.0	AMI without CS	IABP+PCI	PCI	<24 h, AR	4–24	30;180
Prondzinsky 2010 [15]	40	14(73.6)	17(80.9)	62.1	66.1	AMI with CS	IABP+PCI	PCI	13.91±3.06	96	4
Stone 1997 [16]	437	158(74.9)	170(75.2)	64.7	63.7	AMI without CS	IABP+PTCA	PTCA	12	47.9±28.0	30
Thiele 2013 [17]	595	299(66.9)	296(70.6)	UC	UC	AMI with CS	IABP+PCI	PCI	UC	UC	12
Vijayalakshmi 2007 [18]	33	14(82.3)	14(87.5)	39.8	44.7	AMI without CS	IABP+PCI	PCI	7.1±5.5	48	30
Waksman 1993 [19]	45	14(58.3)	15(71.4)	66.8	67.8	AMI with CS	IABP+TT	TT	3	228	30;180

AR:after randomization; CS:cardiogenic shock; IABP:intra-aortic balloon counterpulsation pump; NR, not reported; PTCA:percutaneous transluminal coronary angioplasty; TT: thrombolytic therapy; U, Unclear.

Acorrding to the PRISMA Statement [6], A flow diagram (Figure 1) and a 27-item checklist (Checklist S1) for transparent reporting of a systematic review were used to included in the papper.

### 2. Risk of bias within studies

The 13 selected RCTs provided complete dates of conduct and were free of selective reporting of results or risk of bias. However, three of the RCTs [8], [17], [18] did not clearly describe the methods of randomization or concealment of group allocation (Table 2).

**Table 2:** Methodological quality assessment of the included articles.

Author	Random sequence generation (selection bias)	Allocation concealment(selection bias)	Blinding? (performance bias and detection bias)	Incomplete outcome data addressed?	Free of selective reporting? (reporting bias)	Free of other bias?
Flaherty 1985 [7]	Y	Y	Y	N	Y	Y
Gu 2011 [8]	U	U	Y	Y	Y	Y
Kono 1996 [9]	Y	Y	N	Y	Y	Y
Li 2007 [10]	Y	Y	Y	Y	Y	Y
Ohman 1994 [11]	Y	Y	Y	Y	Y	Y
Ohman 2005 [12]	Y	Y	Y	Y	Y	N
Patel 2011 [13]	Y	Y	Y	Y	Y	Y
Perera 2010 [14]	Y	Y	Y	Y	Y	Y
Prondzinsky 2010 [15]	Y	Y	N	Y	Y	Y
Stone 1997 [16]	Y	Y	Y	Y	N	Y
Thiele 2013 [17]	Y	Y	N	Y	Y	Y
Vijayalakshmi 2007 [18]	U	U	N	Y	Y	Y
Waksman 1993 [19]	U	U	N	Y	Y	Y

N:No; U:Unclear; Y:Yes.

### 3. Mortality within 2 months

**Figure 2:**
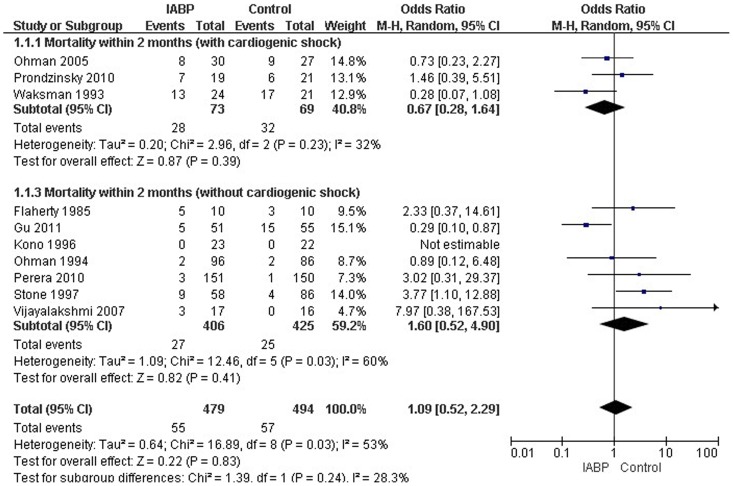
Forest plot of studies evaluating curative effect of IABP in mortality within 2 months.

Ten trials with a total of 973 AMI patients reported 2-month mortality. The *I* ^2^ statistic showed that study heterogeneity was significant (*p* = 0.03, *I* ^2^ = 53%), and thus the random-effects model of DerSimonian and Laird was selected to analyze the data. There was no significant difference in 2-month mortality between the IABP group and the control group (three RCTs enrolled AMI patients with CS (OR = 0.67, 95% CI: 0.28–1.64; *p* = 0.39). Seven RCTs enrolled AMI without CS (OR = 1.60, 95% CI: 0.52–4.90; *p* = 0.41.). (Figure 2).

### 4. Mortality within 6–12 months

**Figure 3:**
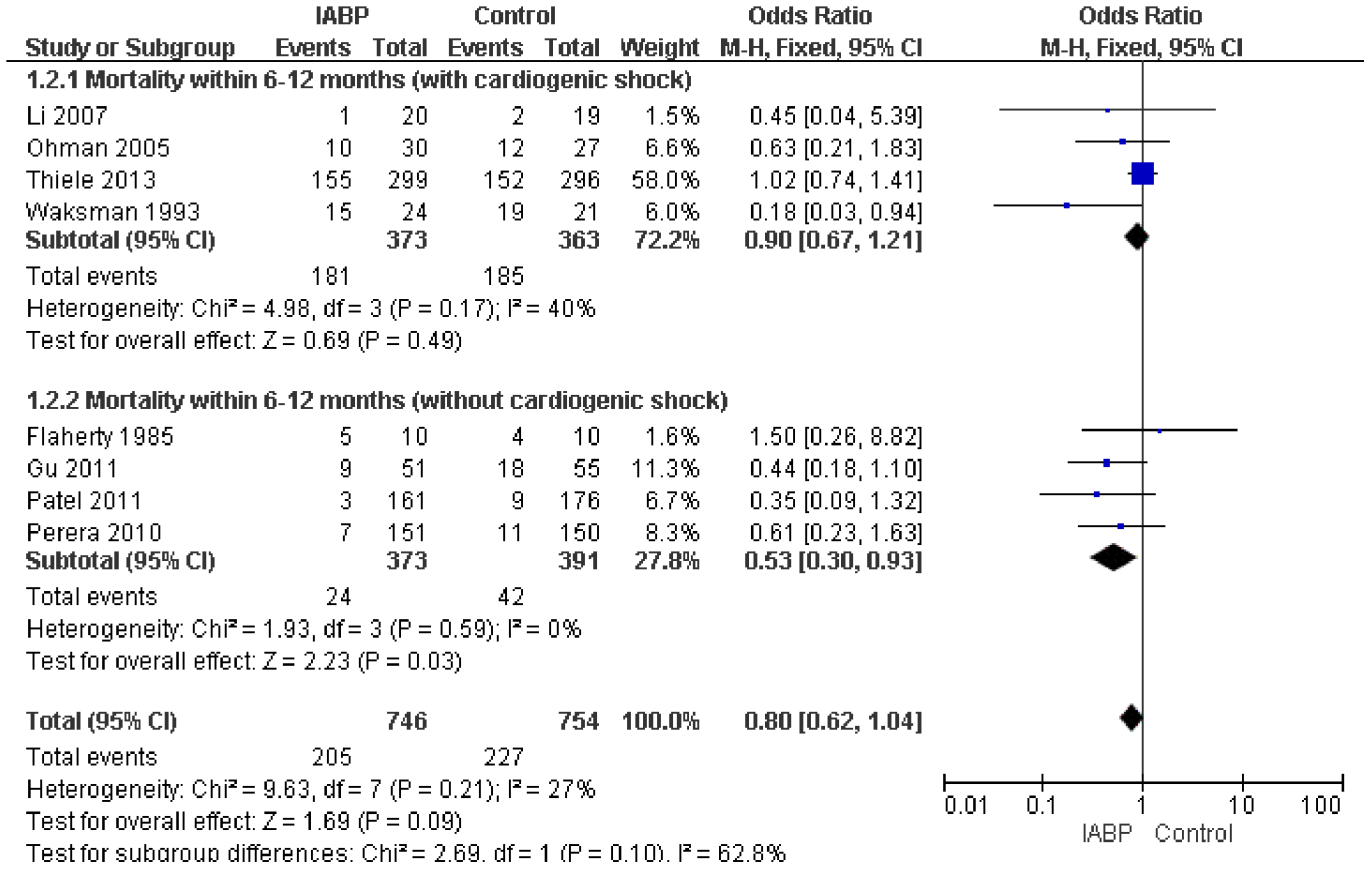
Forest plot of studies evaluating curative effect of IABP in mortality within 6–12 months.

**Figure 4:**
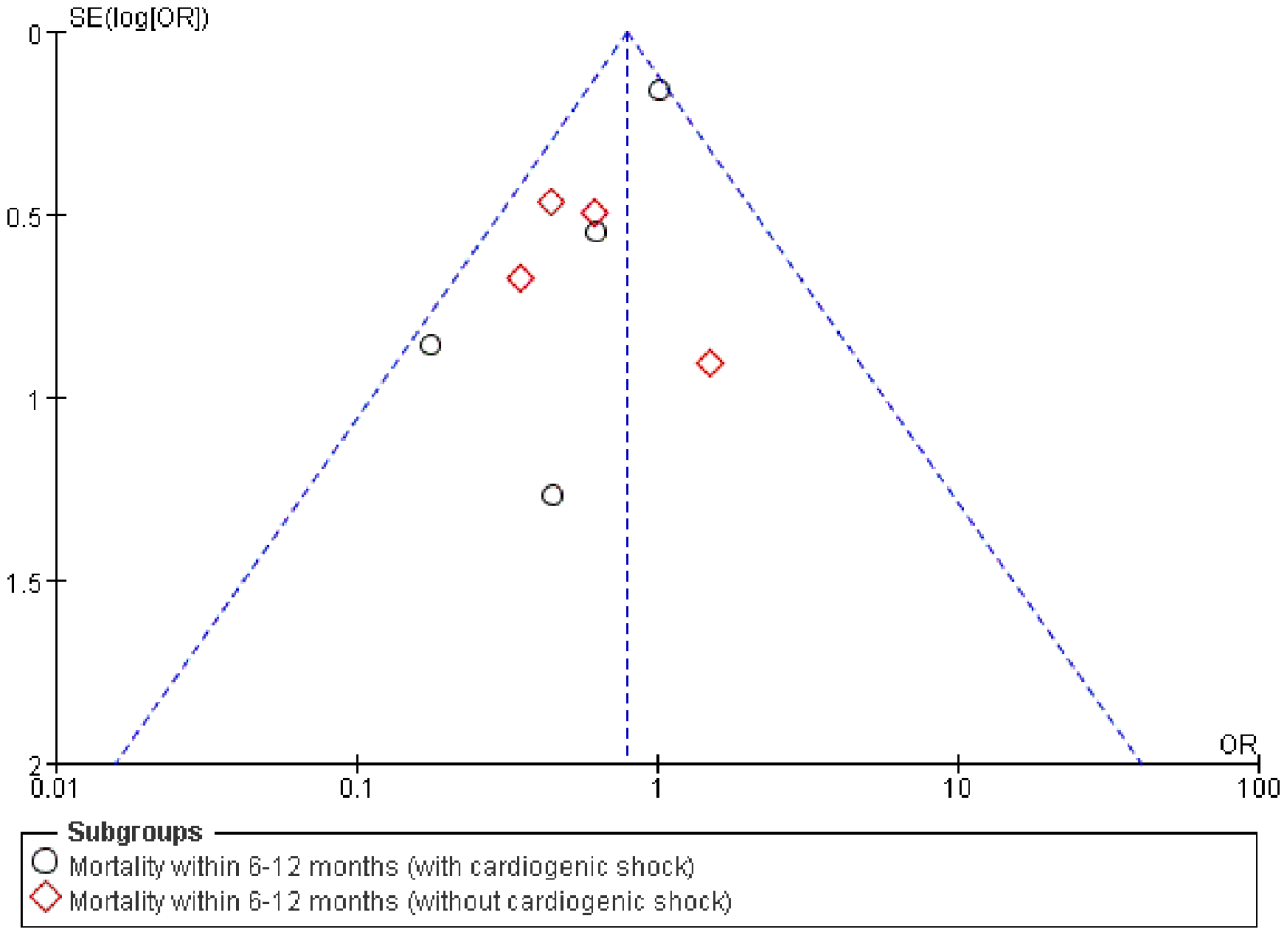
Funnel plot of mortality within 6–12 months.

Seven trials, with a total sample size of 1500, reported 6–12-month mortality. The study results did not show significant heterogeneity (P = 0.21, *I* ^2^ = 27%), and thus the fixed-effects Mantel–Haenzel model was used for data analysis. The 6-month mortality in the IABP group was not significantly lower than in the control group in the four RCTs that enrolled AMI patients with CS (OR = 0.90, 95% CI: 0.67–1.21; *p* = 0.49). But in the four that enrolled AMI patients without CS, the data showed opposite conclusion(OR = 0.53, 95% CI: 0.30–0.93; *p* = 0.03). (Figure 3). The funnel diagram was proximally symmetrical, which indicated no publication bias (Figure 4).

### 5. Bleeding events

**Figure 5:**
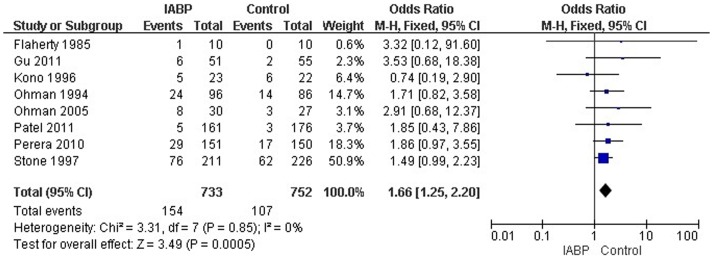
Forest plot of studies evaluating curative effect of IABP in bleeding events.

A total of eight RCTs with a total sample size of 1485 reported bleeding events. The study results did not show significant heterogeneity (*p* = 0.85, *I* ^2^ = 0%) and indicated that the risk of bleeding occurring in IABP patients in IABP was higher than in the control group (OR = 1.66, 95% CI: 1.25–2.20; *p*<0.01) (Figure 5).

## Discussion

This meta-analysis of 12 RCTs investigating treatment of AMI by IABP indicated that (1)IABP can not reduce within 2 months and 6–12 months mortality of AMI patients with cardiac shock (CS), as well as within 2 months mortality of AMI patients without CS, (2)but can reduce 6–12 months mortality of AMI patients without CS. (3)However, IABP increased the risk of bleeding.

Existing research shows that IABP increases blood supply to the coronary artery and the brain, reduces afterload, and eventually decreases cardiac oxygen consumption [1], [2]. It can thus be considered as an adjuvant method for treatment of AMI. The difference in results for short- (1–2 months) and long-term (6–12 months) follow-up indicates that treatment of AMI with IABP may be associated with some slow, compensatory mechanism of myocardial repair, but the specific mechanism is still not clear. A study by Chang [20], showed that in 2 months, the granulation scar tissue in the infarct area changed into connective tissue. In 2–3 months, the infarct area changed into a complete, acellular fibrous scar. These changes in scar tissue are consistent with the effect of IABP on mortality in this meta-analysis and suggest that IABP treatment may be affected by some unknown factors associated with scar formation. More experiments should be conducted to determine the mechanism. Although IABP can reduce AMI patient mortality in the first 6–12 months, it also increases the risk of bleeding. This mechanism is also not clear, thus further studies are needed.

Since 1980s, IABP is widely used in clinic. AMI is commonly seen in internal medicine, and about 7%∼10% of AMI patients with CS has high mortality rate, through timely and effective treatment is performed. Coronary artery recanalization is the key to treat AMI, but in theory IABP can be used to better ensure sufficient blood supply and improve cardiac function of patients so as to save more myocardium and eventually further reduce the adverse consequence of AMI for patients. But, in the process of actual clinical research, the role of IABP is still controversial.

SHOCK registered study [21] and NRMI registered study [22] confirm that application of IABP obviously benefits patients. SHOCK research retrospectively analyzes 856 patients AMI combined with cardiac shock and shows that mortality rate in the hospital of IABP group is better than that of non IABP group (50% vs72%, P<0.0001). NRMI study evaluates American 23180 patients with AMI combined with cardiac shock registered, with total mortality of 70%; 31% of the patients are undergone IABP; among patients with venous thrombolysis, the application of IABP is significantly correlated to case fatality rate (67% vs49%, P<0.05); thrombolysis combined with IABP treatment can reduce the risk of death by 18% (OR0.82; 95%CI 0.72–0.93), but this study also puts forward that application of IABP is not obviously effective in emergency angioplasty(45% vs 47%).

Zeymer [23] retrospectively analyzed 653 cases of patients with acute STEMI and non acute STEMI combined with cardiac shock in 176 European medical centers from May, 2005 to April, 2008, showing only 25% of the patients with IABP in PCI surgery, and there was no significant difference in survival rate between IABP group and non IABP group(OR 1.47; 95% CI 0.97–2.21, P = 0.07). Zeymer [24] carried out a retrospective analysis on 55008 cases of ACS patients (1913 cases combined with shock)with PCI treatment from January, 2006 to December, 2011, among of whom, 487 cases were undergone IABP treatment. The mortality in the hospital for patients with IABP and without IABP was 43.5% and 37.4%, respectively (P = 0.0004), showing the application of IABP is obviously associated with higher fatality rate(OR 1.45,95%CI 1.15–1.84).

Valk [25] retrospectively analyzed 437 AMI patients with IABP from 1999 to 2004 (1990–2004 as the first stage, 1995–1999 as the second stage and 2000–2004 as the third stage), whose results showed that the amount of shock patients in three stages was not different; with the increase of ABP treatment, 30 d case fatality rate was reduced to 26% from 41%; about half of the patients still alive after ten years' follow-up.

In this meta-analysis, major adverse cardiac events (MACEs) were not analyzed because the details of relevant events have not been reported in most published studies. A retrospective study [26] including 1490 AMI patients showed that the occurrence of MACEs in AMI patients with CS was significantly reduced by treatment with IABP (14.5% in the IABP group vs. 35.1% in the control group, *p* = 0.009), and that IABP was also beneficial in AMI patients with decreased left ventricular function. Barron et al. [27] carry out a NRMI-2 study involved 23,180 AMI patients with CS, of whom, 7268 patients were performed with IABP treatment. The result shows that the mortality rate of patients with IABP treatment is significantly lower than that of patients with thrombolytic therapy (67% vs49%). Unfortunately, most of the published studies were not RCTs. Besides, some studies with large sample sizes have yielded conflicting results [16], [28].

Although similar meta-analyses have been published, their conclusions differ from those provided here. In a meta-analysis based on six RCTs (two of which included patients with percutaneous left ventricular assist devices in the control group), no convincing benefit was observed with IABP therapy in AMI patients with CS [29]. Another meta-analysis concluded that IABP did not reduce mortality in AMI patients without CS, but the data were not divided into subgroups based on follow-up duration [30]. Briefly, those analyses concluded that IABP might not benefit AMI patients. However, in this analysis, we came to the opposite conclusion when the data were stratified by different durations of follow-up. Without analyzing different follow-up time, a meta-analysis showed that IABP can not improve LVEF or reduce the occurrence of angina or infarction on AMI patients without CS [31]. However, IABP reduced the mortality of AMI patients with CS, but it increased the incidence of stroke and bleeding.

The present study differs from similar, previously published meta-analyses in that (1) it includes the most recent RCTs; (2) follow-up time was divided into two periods so that the impact of follow-up time can be assessed; and (3) the AMI patients were divided into subgroups with and without CS, thus making it easier to analyze the mechanism of treatment of AMI with IABP.

The present analysis also has certain limitations. (1) The basic therapies for the patients included in the selected studies were different. (2) Both the start-time and duration of IABP differed in the selected studies. (3) The sample sizes of some of the RCTs were relatively small. (4) The quality of some studies was relatively low. (5) The follow-up time in most studies was not sufficiently long. (6) MACEs were not analyzed statistically. To solve these problems, more high-quality clinical studies with longer follow-up time and more detailed records are needed. To confirm the curative effect and safety of IABP in AMI patients, more rigorous studies with larger sample sizes, longer follow-up time and more detailed records should be conducted.

## Conclusions

IABP can not reduce within 2 months and 6–12 months mortality of AMI patients with CS, as well as within 2 months mortality of AMI patients without CS, but can reduce 6–12 months mortality of AMI patients without CS. In addition, IABP can increase the risk of bleeding.

## Supporting Information

Checklist S1 **PRISMA checklist.**
(DOC)

## References

[1] Kern MJ, Aguirre F, Bach R, Donohe T, Siegel R, et al (1993) Augmentation of coronary blood flow by intra-aortic balloon pumping in patients after coronary angioplasty. Circulation 87: 500–511. 8425297 10.1161/01.cir.87.2.500

[2] Kantrowitz A, Tjønneland S, Freed PS, Phillips SJ, Butner AN, et al (1968) Initial clinical experience with intraaortic balloon pumping in cardiogenic shock. JAMA 203: 113–118. 5694059

[3] Anderson JL, Adams CD, Antman EM, Bridges CR, Califf RM, et al (2011) ACC/AHA Practice Guideline.2011 ACCF/AHA focused update incorporated into the ACC/AHA 2007 Guidelines for the management of patients with unstable angina/Non–ST-Elevation myocardial infarction. a report of the American College of Cardiology Foundation/American Heart Association Task Force on Practice Guidelines. Circulation 123: e426–e579. 21444888 10.1161/CIR.0b013e318212bb8b

[4] Higgins JP, Thompson SG, Deeks JJ, Altman DG (2003) Measuring inconsistency in meta-analyses. BMJ 327: 557–560. 12958120 10.1136/bmj.327.7414.557 PMC192859

[5] Sterne JA, Egger M, Smith GD (2001) Systematic reviews in health care: investigating and dealing with publication and other biases in meta-analysis. BMJ 323: 101–105. 11451790 10.1136/bmj.323.7304.101 PMC1120714

[6] Moher D, Liberati A, Tetzlaff J, Altman DG, The PRISMA Group (2009) Preferred reporting items for systematic reviews and Meta-Analyses: The PRISMA Statement. PLoS Med 6(7): e1000097 doi:10.1371/journal.pmed.1000097 19621072 10.1371/journal.pmed.1000097 PMC2707599

[7] Flaherty JT, Becker LC, Weiss JL, Brinker JA, Bulkley BH, et al (1985) Results of a randomized prospective trial of intraaortic balloon counterpulsation and intravenous nitroglycerin in patients with acute myocardial infarction. J Am Coll Cardiol 6: 434–446. 3926848 10.1016/s0735-1097(85)80183-2

[8] Gu J, Hu W, Xiao H, Feng X, Song Z, et al (2011) Prophylactic intra-aortic balloon pump reduces C-reactive protein levels and early mortality in high-risk patients undergoing percutaneous coronary intervention. Acta Cardiol 66: 499–504. 21894807 10.1080/ac.66.4.2126599

[9] Kono T, Morita H, Nishina T, Fujita M, Onaka H, et al (1996) Aortic counterpulsation may improve late patency of the occluded coronary artery in patients with early failure of thrombolytic therapy. J Am Coll Cardiol 28: 876–881. 8837563 10.1016/s0735-1097(96)00240-9

[10] Li J, Xue H, Wang B, Zhang HY, Yin L, et al (2007) Effect of prolonged intra-aortic balloon pumping in patients with cardiogenic shock following acute myocardial infarction. Medical Science Monitor 23: CR270–274. 17534233

[11] Ohman EM, George BS, White CJ, Kern MJ, Gurbel PA, et al (1994) Use of aortic counterpulsation to improve sustained coronary artery patency during acute myocardial infarction. Results of a randomized trial. The Randomized IABP Study Group. Circulation 90: 792–799. 8044950 10.1161/01.cir.90.2.792

[12] Ohman EM, Nanas J, Stomel RJ, Leesa MA, Nielsen DWT, et al (2005) Thrombolysis and counterpulsation to improve survival in myocardial infarction complicated by hypotension and suspected cardiogenic shock or heart failure: results of the TACTICS Trial. Journal of Thrombosis and Thrombolysis 19: 33–39. 15976965 10.1007/s11239-005-0938-0

[13] Patel MR, Smalling RW, Thiele H, Barnhart HX, Zhou Y, et al (2011) Intra-aortic balloon counterpulsation and infarct size in patients with acute anterior myocardial infarction without shock:The CRISP AMI randomized trial. JAMA 306: 1329–1337. 21878431 10.1001/jama.2011.1280

[14] Perera D, Stables R, Thomas M, Booth J, Pitt M, et al (2010) Elective intra-aortic balloon counterpulsation during high-risk percutaneous coronary intervention a randomized controlled trial. JAMA 304: 867–874. 20736470 10.1001/jama.2010.1190

[15] Prondzinsky R, Lemm H, Swyter M, Wegener N, Unverzagt S, et al (2010) Intra-aortic balloon counterpulsation in patients with acute myocardial infarction complicated by cardiogenic shock: the prospective, randomized IABP SHOCK Trial for attenuation of multiorgan dysfunction syndrome. Person-Centered and Experiential Psychotherapies 38: 152–160. 10.1097/CCM.0b013e3181b78671 19770739

[16] Stone GW, Marsalese D, Brodie BR, Griffin JJ, Donohue B, et al (1997) A prospective, randomized evaluation of prophylactic intraaortic balloon counterpulsation in high risk patients with acute myocardial infarction treated with primary angioplasty fn1. J Am Coll Cardiol 29: 1459–1467. 9180105 10.1016/s0735-1097(97)00088-0

[17] Thiele H, Zeymer U, Neuman F, Ferenc M, Olbrich H, et al (2013) Intra-aortic balloon counterpulsation in acute myocardial infarction complicated by cardiogenic shock (IABP-SHOCK II): final 12 month results of a randomised, open-label trial. Lancet 382: 1638–1645. 24011548 10.1016/S0140-6736(13)61783-3

[18] Vijayalakshmi K, Kunadian B, Whittaker VJ, Wright RA, Hall JA, et al (2007) Intra-aortic counterpulsation does not improve coronary flow early after PCI in a high-risk group of patients: observations from a randomized trial to explore its mode of action. J Invasive Cardiol 19: 339–346. 17712202

[19] Waksman R, Weiss AT, Gotsman MS, Hasin Y (1993) Intra-aortic balloon counterpulsation improves survival in cardiogenic shock complicating acute myocardial infarction. Eur Heart J 14: 71–74. 10.1093/eurheartj/14.1.71 8432295

[20] Chang J; Nair V; Luk A, Butany J (2013) Pathology of myocardial infarction. Diagnostic Histopathology 19: 7–12.

[21] Sanborn TA, Sleeper LA, Bates ER, Jacobs AK, Boland J, et al.. (2000) Impact of thrombolysis, intra-aortic balloon pump counterpulsation, and their combination in cardiogenic shock complicating acute myocardial infarction: a report from the SHOCK Trial Registry. Should we emergently revascularize Occluded Coronaries for cardiogenic shock? J Am Coll Cardiol 36(3suppl A):1123–1129. 10.1016/s0735-1097(00)00875-5 10985715

[22] Barron HV, Every NR, Parsons LS, Angeja B, Goldberg RJ, et al (2001) The use of intra-aortic balloon counterpulsation in patients with cardiogenic shock complicating acute myocardial infarction: data from the National Registry of Myocardial Infarction 2. Am Heart J 141: 933–939. 11376306 10.1067/mhj.2001.115295

[23] Zeymer U, Bauer T, Hamm C, Zahn R, Weidinger F, et al (2011) Use and impact of intra-aortic balloon pump on mortality in patients with acute myocardial infarction complicated by cardiogenic shock: results of the Euro Heart Survey on PCI. Euro Intervention 7: 437–441. 21764661 10.4244/EIJV7I4A72

[24] Zeymer U, Hochadel M, Hauptmann KE, Wiegand K, Schuhmacher B, et al (2013) Intra-aortic balloon pump in patients with acute myocardial infarction complicated by cardiogenic shock: results of the ALKK-PCI registry. Clin Res Cardiol 102: 223–227. 23179136 10.1007/s00392-012-0523-4

[25] Valk SDA, Cheng JM, den Uil CA, Lagrand WK, van der Ent M, et al (2011) Encouraging survival rates in patients with acute myocardial infarction treated with an intra-aortic balloon pump. Neth Heart J 19: 112–118. 21475411 10.1007/s12471-010-0066-0 PMC3047716

[26] Brodie BR, Stuckey TD, Hansen C, Muncy D (1999) Intra-aortic balloon counterpulsation before primary percutaneous transluminal coronary angioplasty reduces catheterization laboratory events in high-risk patients with acute myocardial infarction. Am J Cardiol 84: 18–23. 10404845 10.1016/s0002-9149(99)00185-x

[27] Barron HV, Every NR, Parsons LS, Angeja B, Goldberg RJ, et al (2001) The use of intra-aortic balloon counterpulsation in patients with cardiogenic shock complicating acute myocardial infarction: Data from the National Registry of Myocardial Infarction 2. Am Heart J 141: 933–939. 11376306 10.1067/mhj.2001.115295

[28] Van't Hof AW, Liem AI, Boer MJ, Hoorntje JC, Suryapranata H, et al (1999) A randomized comparison of intra-aortic balloon pumping after primary coronary angioplasty in high risk patients with acute myocardial infarction. Eur Heart J 20: 659–665. 10208786 10.1053/euhj.1998.1348

[29] Unverzagt S, Machemer MT, Solms A, Thiele H, Burkhoff D, et al.. (2011) Intra-aortic balloon pump counterpulsation (IABP) for myocardial infarction complicated by cardiogenic shock. Cochrane Database Of Systematic Reviews (Online) 7. Available: http://onlinelibrary.wiley.com/doi/10.1002/14651858.CD007398.pub2/abstract. Assessed 19 July 2010. 10.1002/14651858.CD007398.pub2 21735410

[30] Cassese S, Waha A, Ndrepepa G, Ranftl S, King L, et al (2012) Intra-aortic balloon counterpulsation in patients with acute myocardial infarction without cardiogenic shock. A meta-analysis of randomized trials. Am Heart J 164: 58–65. 22795283 10.1016/j.ahj.2012.05.001

[31] Bahekar A, Singh M, Singh S, Bhuriya R, Ahmad K, et al (2012) Cardiovascular outcomes using intra-aortic balloon pump in high-risk acute myocardial infarction with or without cardiogenic shock: A Meta-Analysis. J Cardiovasc Pharmacol Ther 17: 144–156. 10.1177/1074248410395019 21335478

